# The Health-Related Quality of Life for Patients with Myalgic Encephalomyelitis / Chronic Fatigue Syndrome (ME/CFS)

**DOI:** 10.1371/journal.pone.0132421

**Published:** 2015-07-06

**Authors:** Michael Falk Hvidberg, Louise Schouborg Brinth, Anne V. Olesen, Karin D. Petersen, Lars Ehlers

**Affiliations:** 1 Danish Center for Healthcare Improvements, Aalborg University, Aalborg, Denmark; 2 Coordinating Research-unit, Frederiksberg Hospital, Frederiksberg, Denmark; San Raffaele Scientific Institute, ITALY

## Abstract

**Introduction:**

Myalgic encephalomyelitis (ME)/chronic fatigue syndrome (CFS) is a common, severe condition affecting 0.2 to 0.4 per cent of the population. Even so, no recent international EQ-5D based health-related quality of life (HRQoL) estimates exist for ME/CFS patients. The main purpose of this study was to estimate HRQoL scores using the EQ-5D-3L with Danish time trade-off tariffs. Secondary, the aims were to explore whether the results are not influenced by other conditions using regression, to compare the estimates to 20 other conditions and finally to present ME/CFS patient characteristics for use in clinical practice.

**Material and methods:**

All members of the Danish ME/CFS Patient Association in 2013 (n=319) were asked to fill out a questionnaire including the EQ-5D-3L. From these, 105 ME/CFS patients were identified and gave valid responses. Unadjusted EQ-5D-3L means were calculated and compared to the population mean as well as to the mean of 20 other conditions. Furthermore, adjusted estimates were calculated using ordinary least squares (OLS) regression, adjusting for gender, age, education, and co-morbidity of 18 self-reported conditions. Data from the North Denmark Health Profile 2010 was used as population reference in the regression analysis (n=23,392).

**Results:**

The unadjusted EQ-5D-3L mean of ME/CFS was 0.47 [0.41–0.53] compared to a population mean of 0.85 [0.84–0.86]. The OLS regression estimated a disutility of -0.29 [-0.21;-0.34] for ME/CFS patients in this study. The characteristics of ME/CFS patients are different from the population with respect to gender, relationship, employment etc.

**Conclusion:**

The EQ-5D-3L-based HRQoL of ME/CFS is significantly lower than the population mean and the lowest of all the compared conditions. The adjusted analysis confirms that poor HRQoL of ME/CFS is distinctly different from and not a proxy of the other included conditions. However, further studies are needed to exclude the possible selection bias of the current study.

## Introduction

Myalgic encephalomyelitis (ME) / chronic fatigue syndrome (CFS) is a condition reported worldwide and leads to extensive debility and suffering [1,2]. The condition can be as disabling as multiple sclerosis, rheumatoid arthritis, systemic lupus erythematosus and congestive heart failure [3]. It is a controversial and much debated illness with unclear medical aetiology, making diagnosis and treatment challenging. There are different diagnostic criteria for ME/CFS, but a general definition of the diagnosis is the presence of over six months of fatigue, post-exertion malaise and a restriction of the ability of those affected to sustain previous levels of social, work and leisure activities [4]. Other common symptoms range from pain to cognitive impairment and more [2].

Several different aetiologies for CFS/ME have been investigated—neurological, immunological, endocrine, psychiatric, genetic, and infectious—but, so far, the aetiology cannot be fully explained [4,5], although new and promising explanations and treatments are emerging [6].

The condition is considered common, affecting from 0.2 to 0.4 per cent of the population [3]. In a Danish context this equals between 11,000 and 22,000 patients. However, the prevalence is debated and varies with the diagnostic criteria applied and some studies suggest a prevalence as high as four to eight per cent, yielding an estimated number of patients in Denmark as high as 125,000 patients [7].

Even though the condition is disabling, severe and common, there is, to the authors’ knowledge, no Danish or recent international health-related quality of life (HRQol) EQ-5D-3L score estimated (see later section for description of the EQ-5D-3L).

A recent literature review of 2012 found only one, older Scottish study from 1999 using the EQ-5D-3L [8,9]. Also, no adjusted ordinary least squares (OLS) regression estimates based on the EQ-5D has so far been presented calculating the pure reduced HRQoL (disutility) of ME/CFS distinct (adjusted) from other conditions. Hence, there are no updated nor any Danish EQ-5D-3L estimates, which is the preferred measure in health economics assessments [10], for use in health-care planning, even though the condition causes a substantial burden for both patients and society.

The aim of this study is to estimate HRQoL scores using the EQ-5D-3L for ME/CFS patients with Danish national time trade trade-off tariffs [11]. EQ-5D-3L estimates of ME/CFS adjusted for gender, age, education, and co-morbidity using OLS-regression are also presented and investigated. The OLS regression is expected to shed light on the characteristics of the condition by estimating the pure HRQoL of ME/CFS controlled for other conditions. This will reveal whether the condition’s impact on HRQoL is actually separate from other conditions statistically, or whether the impact of ME/CFS on the HRQoL is merely a proxy for other conditions—and thus indirectly contribute to the debate regarding pathophysiology and aetiology of the condition. Moreover, the estimates of ME-/CFS patients will be compared to 20 other conditions and patient characteristics of ME/CFS are also explored.

## Material and Methods

### Health-related quality of life—the EQ-5D

The EQ-5D-3L is a generic, self-reported questionnaire with five dimensions: 1) mobility; 2) self-care; 3) usual activities; 4) pain/discomfort; and 5) anxiety/depression [12]. Each dimension has three levels of severity, ranging from level one, indicating no problems in the dimension, to level three, signifying extreme problems.

A total of 243 combinations convert into a linear scale ranging from -0.624 to 1.000 –a so called index *utility-score* (where negative values are conditions considered worse than death). EQ-5D-3L can be used both as a profile score of a group and as a single index score of a person’s HRQoL. The main difference from other generic HRQoL measures is that the scale is preference-based, meaning that the 243 response-combinations are valued by a representative population unique to each country. Thus, the scores reflect the countries’ values of the five dimensions and 243 combinations—not those of clinicians or scientists. The single index score in this study was then obtained by incorporating Danish population preference weights (time trade trade-off tariffs) [11].

Since the measure is generic, it is ideal to use for a comparison across conditions or other groups. The EQ-5D scale is the main and dependent variable of use.

### The samples and characteristics—and inclusion criteria

The study was based on two Danish cross-sectional samples. The first sample consisted of 112 respondents from the national ME/CFS patient association sampled in 2013/2014. Of the 112 respondents, 7 had missing values regarding the question of having ME/CFS and were excluded for the complete case analysis. Thus, of the 105 patients having self-reported ME/CFS, 2 had missing answers on the EQ-5D; 103 respondents were included in estimating the unadjusted HRQoL of ME/CFS patients.

The second sample was the North Denmark Health Profile Survey (NDHPS), a randomized population sample, stratified by municipalities with 23,392 respondents sampled in 2010 [13,14]. The second sample was used as a population reference in the different analyses, including the adjusted OLS-regression analysis.

Both samples comprised the EQ-5D-3L questionnaire and questions about age, gender, 18 broadly defined self-reported conditions (see variables section for details), long-term illness, education, life satisfaction, residence, employment status, and family status. Questions regarding having the ME/CFS condition were only asked in the ME/CFS survey.

There was a three to four year time difference between the ME/CFS (2013/2014) sample and the reference population sample (2010). The time difference between the two samples was not expected to have any significant impact on the results, since neither the EQ-5D-3L mean nor response distributions of the five dimensions differ significantly from the more recently published Health Profile Survey of 2013 [15].

#### Data collection

The data collection concerning the ME/CFS sample was carried out in cooperation with the Danish ME/CFS association. For comparison, the five-page self-administered questionnaire consisted of the EQ-5D-3L and selected questions (see S1 File) identical to those from the Danish National Health Profiles. The Danish ME/CFS association contacted *all* their national members twice, once in the autumn of 2013 and once in the winter of 2014. The survey was, likewise, advertised twice in a magazine for members and on the association’s Facebook page. All of the 319 members were contacted, asked to fill out the questionnaire and return it by letter. However, not all members had ME/CFS since members also include family of patients and supporters who do not have ME/CFS. This makes the response rate imprecise and difficult to estimate, since only ME/CFS patients have returned the questionnaire. Nonetheless, based on 112 answers and the 319 included members, the response rate was approximately 35 per cent—thus not surprisingly reflecting the severity of the condition.

Data collection for the reference population sample was carried out from February to April 2010 by the North Denmark Region. The response rate was 65.5 per cent. The details of the data collection can be read in references [13,14].

#### Variables

The following variables were used:

Education: the educational level of the survey participant was measured as her/his highest self-reported attained education at survey response time, divided into four categories: 1) student/in training or with no qualification; 2) skilled, baccalaureate, or shorter education; 3) medium education (bachelor degree or equivalent); and 4) higher education (master degree or higher).

Employment status: employment status was measured as the current affiliation to the work market at survey-response time. Employment status was categorized into six groups: 1) working (full / part-time); 2) unemployed / benefits; 3) sick leave / long-term illness; 4) disability pensioners; 5) age-related retirement / pension; 6) other/students etcetera.

Relationship: Married / cohabiting or no partner (Yes/no) at time of response.

*Long-term illness*: Self-reported by the question: ‘Do you have any long-term illness, long-term after effects of injuries, disability or other long-term illness?’ Yes/no at time of response.

Self-reported conditions: In the *adjusted* regression analysis, each of the following 18 self-reported conditions is presented dichotomously (having/not having): ME/CFS, stroke, long-term mental illness, short-term mental illness, herniated disc or other back conditions, osteoporosis, heart attack, osteoarthritis, migraine or frequent headaches, rheumatoid arthritis, cancer (any), lung diseases: chronic bronchitis, COPH etc., diabetes, heart attack, cataracts, allergy, high blood pressure and tinnitus.

Morbidity: the mean number of the 18 self-reported conditions.

Age: age at time of survey-response for both samples.

Gender: man/woman.

#### Statistical analysis

The following statistical analysis and presentations were conducted:

- Frequencies and percentages of the selected socio-economic and morbidity variables of the two samples for characterization and comparison.
- Raw frequencies and percentages of the EQ-5D-3L profiles (the five dimensions) for each sample, and proportions significance test for difference.
- Unadjusted means, medians, and 95% confidence intervals of the EQ-5D-3L index scores of ME/CFS—and 20 physician-reported conditions from a previous study for comparison with ME/CFS.
- OLS regression disutility estimates of the EQ-5D-3L index scores adjusted for gender, age, education, and co-morbidity of 18 self-reported conditions.
- Ratios between the ME/CFS EQ-5D-3L means/medians and population EQ-5D-3L means/medians.

For the regression analysis, the ME/CFS and population samples were combined. This approach enabled an adjusted analysis by using the population sample as a ‘reference’. This was possible since both samples contained identical questions.

The ratios were calculated as the EQ-5D-3L mean and median of ME/CFS patients divided by the EQ-5D-3L mean and median of the population. The ratios enable comparison to other studies in the discussion section, and thus express the relative difference between ME/CFS patients and the population. Regarding interpretation of the estimate, the ratio reflects the ME/CFS HRQoL proportion of the population mean. A smaller ratio value signifies a smaller ME/CFS HRQoL proportion to the population mean/median—and thus a larger distance from the ME/CFS mean/median to the population mean/median. The ratios make comparisons more transparent across different national EQ-5D tariffs, different HRQoL measures such as the SF-36, and comparisons over time. Since the EQ-5D population mean is seen to vary over time, so too might that of the ME/CFS population. We intuitively expect the ratios to be *fairly* constant across different national EQ-5D tariffs, other HRQol measures and across time even if the population and condition means vary. Hence, the use of ratios enables us to compare the current study’s results to the few other and older existing studies in pursuit of evaluating the validity of our results.

Data management and data processing will be done in SAS 9.4 using the proc freq, proc surveyfreq, proc surveymean and proc surveyreg. Imputation and the OLS regression based on the imputed values will be done in STATA MP version 12.1, using “MI impute” and “MI estimate regress” with the “svy” option for incorporation of weights.

#### Missing values

Regarding the ME/CFS sample, 2 (1.9 per cent) out of the 105 ME/CFS patients had missed two of the five EQ-5D-3L questions, pain/discomfort and anxiety/depression (one for each person). The missing values will be imputed for each dimension separately. The imputed values did not change the EQ-5D mean or median scores significantly from a complete case approach. However, for both samples the main challenge was the accumulated missing values due to the covariables. For the ME/CFS sample, the covariables of use (gender, age, education and the 18 self-reported conditions) had a total of 21.0 per cent missing values. Hence, a full imputation using chained equations in STATA will be done for all covariables for use in the OLS regression analysis [16]–with the exception of the heart attack variable, where missing values will be set to 0 (no ME/CFS respondents had suffered a heart attack and the imputation cannot compute without at least one of each value).

As for the larger reference population sample, 440 (1.8 per cent) of a total of 23,392 respondents had missing values from the EQ-5D questions. An extreme case strategy analysis of the EQ-5D missing values did not show significant impact on the EQ-5D-3L sample means when imputing different extreme values. A complete-case regression analysis had a total of accumulated missing values of 24.5 per cent. Hence, a full imputation of all missing values will also be done for each of the five EQ-5D questions, gender, age, education and the 18 self-reported conditions, using chained equations with survey weights for the population reference sample.

The chained imputation will be done separately for the two samples in case of different missing mechanisms. The two samples are afterwards combined and analysed. The sensitivity analysis showed that none of the imputation models changed the overall results or majority of the disutility estimates significantly from the complete-case approach. However, we did gain several reduced standard errors using imputed values.

The *unadjusted* results and sample characteristics will be presented using raw responses without imputed values for both samples. All *adjusted* regression analyses will be presented with the imputed values based on 20 imputed data sets as recommended as best practice [17,18]; the “seed” was set to 1978.

#### Geographic representativeness, standardization, and weighting

As shown in Table 1, there were differences of geographic distribution between the ME/CFS sample distribution and the real population distribution regarding Central Denmark Region and Region Zealand, while the distribution of the other regions was fairly consistent with the population average. Also, while ME/CFS patient HRQol does not vary significantly across countries according to other studies [19], we did not expect it to vary significantly between regions within one country. Hence, there is no apparent reason for weighting geographically within the ME/CFS sample; in consequence no differentiated weighting is done for the ME-sample. Furthermore, since no other socio-demographic information exits on the “true” Danish ME/CFS population to estimate weights from, it is not possible to generate differentiated unique weights accounting for non-response.

**Table 1:** ME/CFS sample compared to the full population geographically (n,%). Complete cases.

	ME/CFS sample 2013–2014	Full population 1/1/2013 (Statistics Denmark, dst.dk)
**North Denmark Region**	11 (10.5%)	580.272 (10.4%)
**Central Denmark Region**	13 (12.3%)	1.272.510 (22.7%)
**Region of Southern Denmark**	24 (22.9%)	1.201.419 (21.4%)
**Region Zealand**	24 (22.9%)	816.359 (14.6%)
**Capital Region of Denmark**	33 (31.4%)	1.731.976 (30.9%)

Conversely, the population reference sample was standardized by weights to equal the national average population in respect of gender, age, and education. The weights also took account of non-response; and were pre-computed by Statistics Denmark [14]. The use of standardization weights was done to make up for the fact that we did not have access to a national representative sample with the EQ-5D-3L to be used as reference. The reference population sample was then used as a substitute for a ‘national reference’ in the adjusted regression analysis. Unadjusted mean and median estimates for ME/CFS patients were not weighted, while all computed statistics using the population reference sample were done using the standardized weights.

However, when the two samples are combined and the standardized weight of the population references sample is applied, the ME-sample will be excluded in the regression analyses if no weights values exist for the ME/CFS patients. Thus, the same uniform value of 1/23,504 is inserted into the weight for all the 112 ME/CFS patients. The uniform weight values sum to the full combined sample size of 23,504 respondents (but do naturally not take non-response or other into account). The solution is not optimal, but is the best possible solution when pooling a large, weighting dataset with a small, un-weighted dataset. The solution is tested and compared to a non-weighted model, where there were no differences in respect of estimates and results.

#### Ethical considerations

Both surveys had cover letters explaining the purpose of the surveys, voluntariness of participation, that all responses would be treated confidentially and according to the Danish Data Protection Laws and that all the responses would be published without reference to any individuals. Furthermore, the ME/CFS survey was anonymous with no possible identifier, while the National Health Profile did have a unique identifier for the research purposes of merging data with scientific national registries. This was approved by the Danish Data Protection Agency, and also included in the survey cover letter. Finally, the North Denmark Region legal department approved the current study on behalf of the Danish Data Protection Agency.

## Results

ME/CFS patient characteristics are different to those of the population in several aspects as seen in Table 2. First of all, 87.2 per cent of ME/CFS patients are women. Furthermore, the patients have 20 percentage points fewer relationships than the average population, much higher unemployment, with only 7.6 per cent employed (population 52.2 per cent) and a higher proportion of disability pensioners– 52.2 per cent (population 5.2 per cent) In addition, ME/CFS patients in this study have 2.9 numbers of chronic illnesses on average (co-morbidity)–more than twice as high as the general population. Self-reported long-term illness, as defined previously, is as high as 89.0 per cent, contrary to a population estimate of approximately one-third. On the other hand, ME/CFS patients are higher educated than the reference population.

**Table 2:** Respondent characteristics in the two source data samples. Complete cases.

	ME/CFS sample in Denmark	North Denmark Health profile reference sample (NDHPS)
**Collection year**	*2013–2014*	2010
**Initial sample/invited (n)**	319	35,700
**Valid responses (n, %)**	112, hereof 105 with ME/CFS (33–45%)	23,392 (65.5%)
**Missing EQ-5D-3L responses (n, %)**	2 (1.9%)	440 (1.8%)
**Selection**	All members and ages of the ME/CFS Association Denmark. Geographically the whole of Denmark	Random selection North Denmark Region. Age 16+
**Morbidity: Mean number of illness (SD)**	2.9 (1.8)	1.3 (1.5)
**Long term illness (n, %)**	92 (88.5%)	7.166 (31.6%)
**Mean age (SD)**	50.3 years (13.6)	47.6 years (18.2)
**Women (n,%)**	91 (86.7%)	11.764 (50.0%)
**Education (n,%)**:		
**- Student/in training or no qualification**	11 (10.8%)	7.007 (30.4%)
**- Skilled, baccalaureate, or shorter education**	38 (37.3%)	10.671 (45.8%)
**- Medium education (bachelor degree or eq.)**	39 (38.2%)	3.741 (15.9%)
**- Higher education (master degree or higher)**	14 (13.7%)	1.221 (7.9%)
**Employment status (n,%)**		
**Working (full/part-time)**	8 (7.8%)	11.811 (53.2%)
**Unemployed/benefits**	10 (9.7%)	1.070 (6.2%)
**Sick leave / long-term illness**	6 (5.8%)	178 (0.9%)
**Disability pensioners**	54 (52.4%)	1.029 (5.1%)
**Age-related retirement/pension**	12 (11.7%)	5.273 (19.3%)
**Other/students etcetera**	13 (12.6%)	3095 (15.4%)
**Relationship (n, %)**		
**Living with partner or married**	56 (53.3%)	16664 (67.9%)
**Single or living home/with other**	49 (46.7%)	5934 (32.1%)
**Standardized by weight**	No	Yes

**Fig 1:**
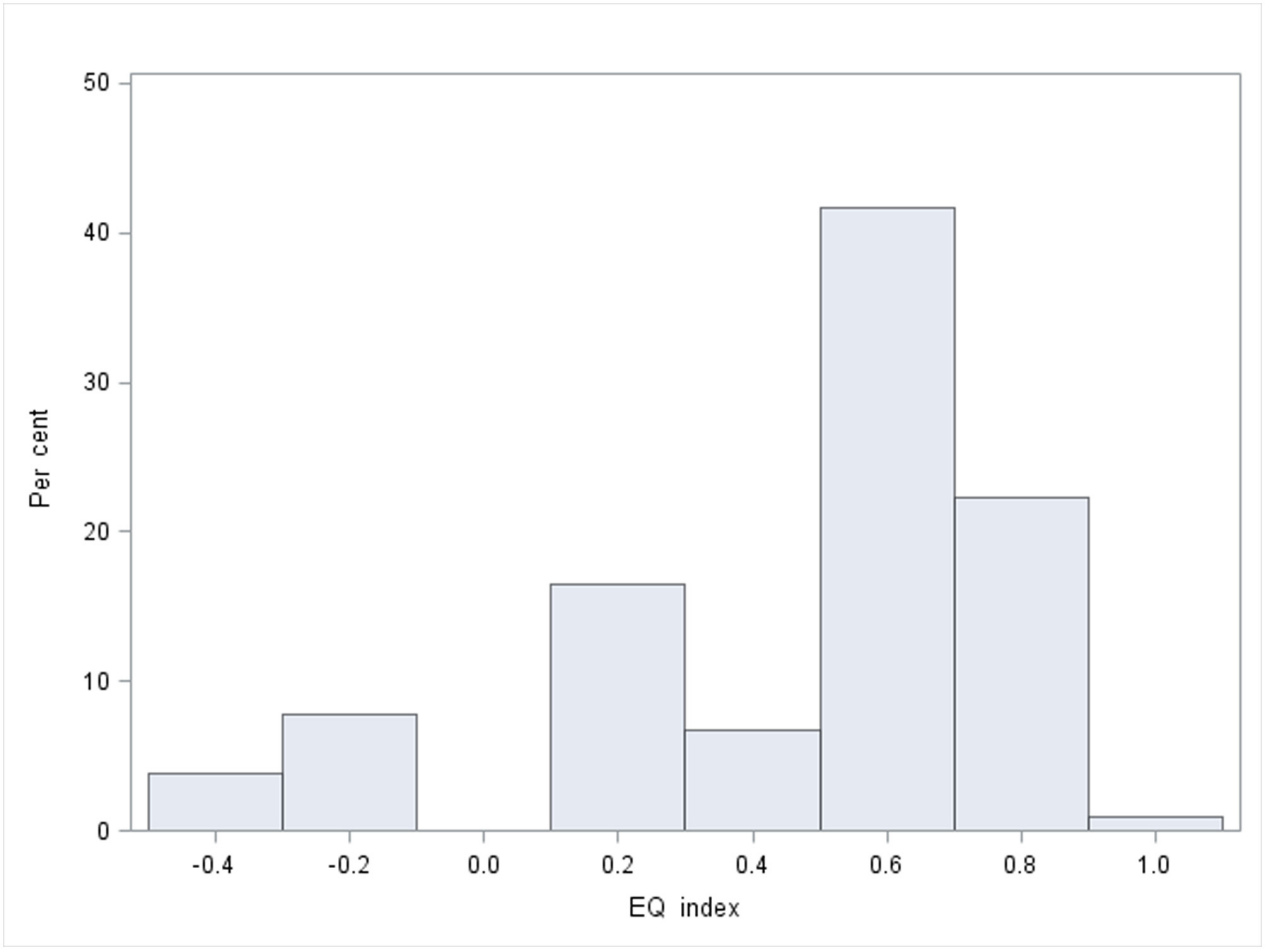
EQ-5D-3L distribution ME/CFS sample.

**Fig 2:**
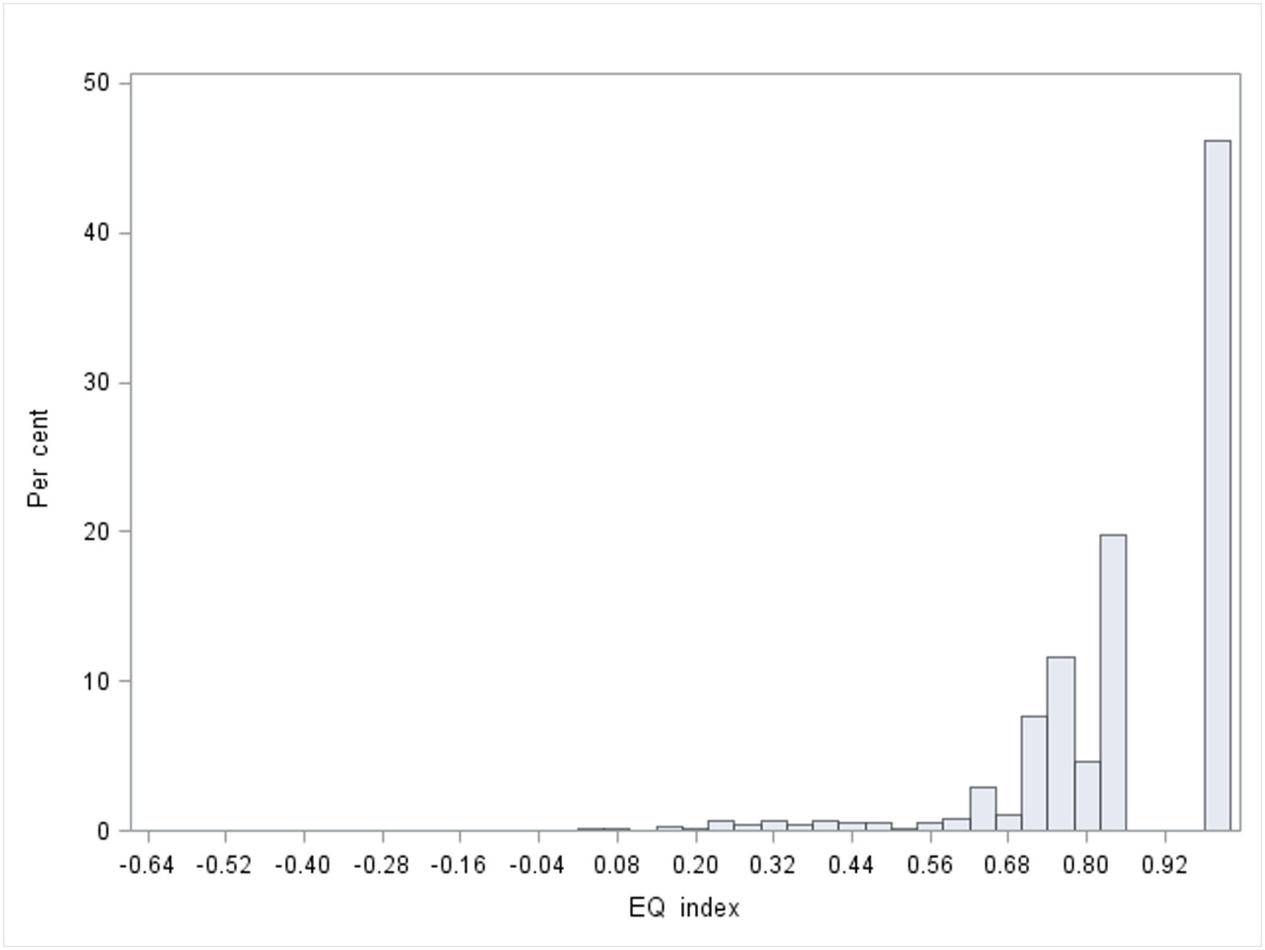
EQ-5D-3L distribution population sample.

The same trends are also seen in respect of the profile scores of the HRQoL, where ME/CFS patients are different in most EQ-5D-3L dimensions compared to the reference population sample (Table 3). The only dimension that is somewhat close to the population responses is anxiety/depression. While the reference population sample responses in the EQ-5D-3L score distributions are much more left skewed, with close to 1 as the most frequent score, the ME/CFS patients most frequent score and centre is around 0.6 (Fig 1 and Fig 2).

**Table 3:** Three level responses on the five EQ-5D-3L dimensions for the two samples (n,%). Complete cases.

	No problems		Some problems		Cannot/extreme problems	
	ME/CFS sample	Population sample	ME/CFS sample	Population sample	ME/CFS sample	Population sample
**Mobility**	16* (14.9%)	19.416 (83.5%)	77* (73.6%)	3.671 (15.8%)	12* (11.5%)	58 (0.3%)
**Self-care**	49* (44.8%)	22.088 (95.0%)	48* (48.3%)	941 (4.4%)	8* (6.9%)	114 (0.6%)
**Usual Activities**	2* (2.3%)	17.583 (75.7%)	49* (46.0%)	4.722 (20.7%)	54*(51.7%)	814 (3.6%)
**Pain/Discomfort**	3* (3.5%)	12.440 (54.8%)	73* (69.8%)	9.883 (41.5%)	28* (26.7%)	784 (3.7%)
**Anxiety /Depression**	65* (66.3%)	18.537 (78.8%)	34* (31.4%)	4.265 (19.6%)	5 (2.3%)	280 (1.6%)

* Significantly different from population sample using 95% confidence intervals for proportions.

In addition, the mean and median EQ-5D-3L utility scores are significantly lower than the population mean and median scores (Table 4). The ratios specify that the HRQoL mean is 55.0 per cent of the population mean and the median HRQoL is 71.8 per cent of the population median. The OLS regression estimates a disutility of -0.29 [-0.21;-0.34] for ME/CFS patients (Table 5), which equals an EQ-5D-3L utility of 0.29 below the population mean of 0.85. The adjusted ME/CFS EQ-5D-3L utility score is then 0.56 (0.85–0.29). Models including interaction terms have also been constructed, but not presented, since no interaction terms changed the overall results. In summary, we only found significant negative interactions with ME/CFS and osteoporosis and osteoarthritis, and positive significant interactions with high blood pressure and education (see S2 File).

**Table 4:** Unadjusted EQ-5D-3L means, median scores and ratios for ME/CFS and population. Complete cases.

	Mean /Median	SE	95% Confidence Intervals (CI)	Ratios
**ME/CFS—mean**	0.469	0.031328	[0.408;0.530]	0.550
**ME/CFS—median**	0.584	0.026863	[0.530;0.637]	0.718
**Population—mean**	0.852	0.001497	[0,849;0.855]	–
**Population—median**	0.824	0.002532	[0.819;0.829]	–

ME/CFS: n = 103. Population: n = 22954.

**Table 5:** OLS regression on EQ-5D-3L adjusted for gender, age, education, and self-reported co-morbidity based on both samples (imputed cases).

	Regression estimates (disutility)	95% CI	n
**ME/CFS**	-0.29**	[-0.35;-0.23]	108
**Short-term mental illness**	-0.14**	[-0.15;-0.13]	2713
**Stroke**	-0.14**	[-0.18;-0.10]	429
**Long-term mental illness**	-0.14**	[-0.16;-0.11]	798
**Herniated disc or other back conditions**	-0.10**	[-0.11;-0.09]	3468
**Osteoarthritis**	-0.07**	[-0.08;-0.07]	5534
**Migraine or frequent headaches**	-0.06**	[-0.07;-0.05]	3714
**Osteoporosis**	-0.06**	[-0.08;-0.04]	1009
**Rheumatoid arthritis**	-0.05**	[-0.06;-0.04]	1640
**Angina pectoris**	-0.04*	[-0.08;-0.01]	796
**Lung diseases: chronic bronchitis, COPH / others**	-0.03**	[-0.05;-0.01]	1407
**Cancer (any)**	-0.02*	[-0.05;0.00]	686
**Diabetes**	-0.02*	[-0.03;0.00]	1564
**Allergy**	-0.01*	[-0.02;0.00]	3834
**High blood pressure**	-0.01*	[-0.02;0.00]	5512
**Cataracts**	0.00	[-0.02;0.02]	1168
**Tinnitus**	0.00	[-0.01;0.01]	2196
**Heart attack**	0.02	[-0.02;0.06]	1024
**Gender, men**	0.01**	[0.00;0.01]	
**Age, continuous (estimate counts each increasing year)**	-0.00**	[0.00;0.00]	
**Education—Student/in training or no qualification, ref**	-	-	
**Education—Skilled, baccalaureate, or shorter education**	0.02**	[0.01;0.02]	
**Education—Medium education (bachelor degree or eq.)**	0.03**	[0.03;0.04]	
**Education—Higher education (master degree or higher)**	0.04**	[0.03;0.05]	
**Intercept**	0.94**	[0.93;0.95]	

** Significant < 0,01

* significant < 0,05. Number of observations in analysis, n = 23.504.

## Discussion

The ME/CFS study population is more disabled and socially marginalized than the average population with regards to the subjects of long-term illness, number of illnesses, proportion of disability pensioners and relationships. The gender and age distribution of the ME/CFS patients are, overall, similar to most other ME/CFS studies [2,20–22], although the mean age of the current study is somewhat older than a few older studies [1,19], as seen in Table 2.

**Fig 3:**
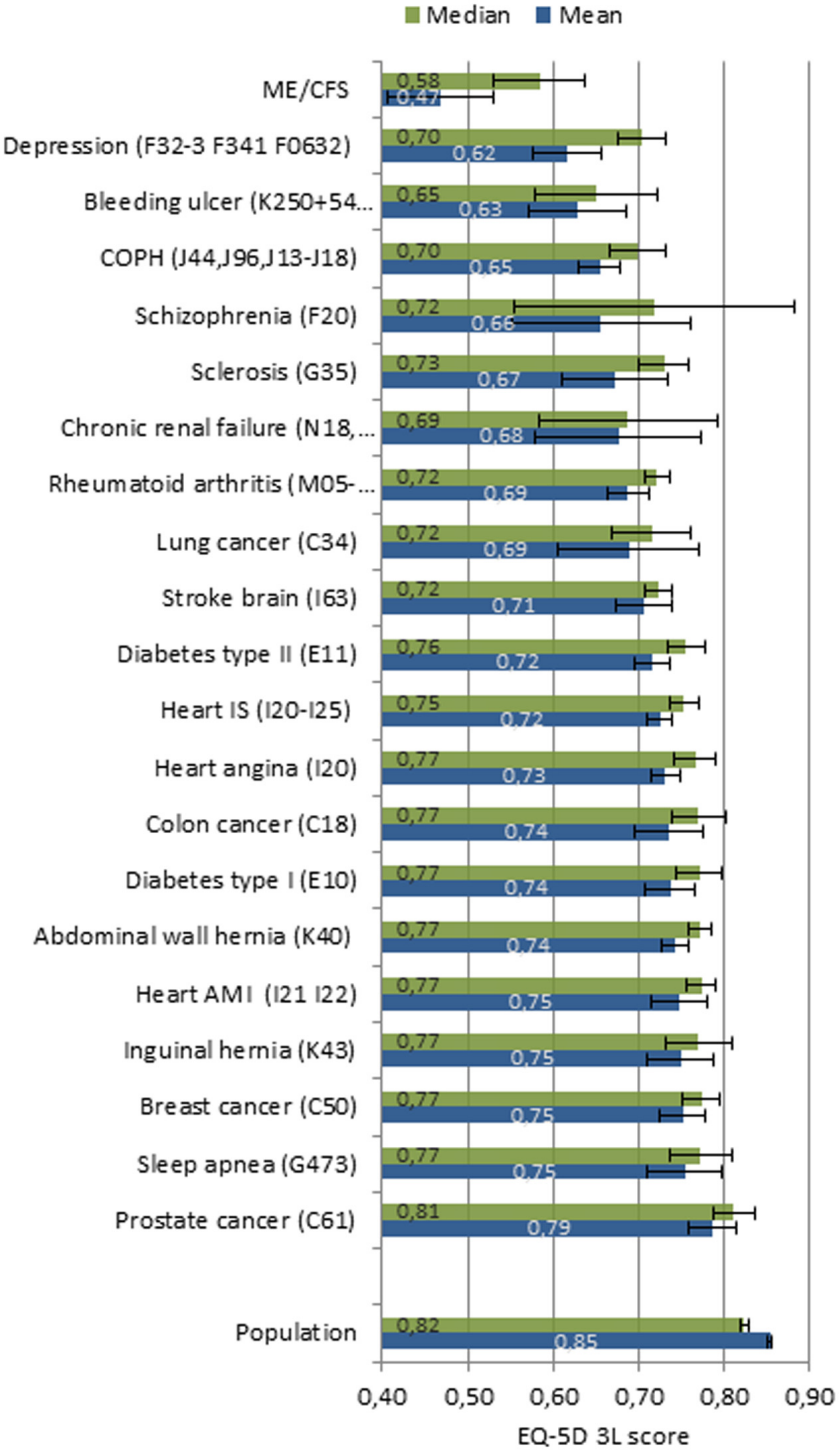
Unadjusted means and medians compared to different conditions.

Based on the present findings, ME/CFS patients in this study have an unadjusted EQ-5D-3L HRQoL utility score of 0.47 and an adjusted one of 0.56. Compared to other conditions shown in Fig 3 from another more recent study [23], the ME/CFS patients of the current study have the lowest, unadjusted EQ-5D-3L measured HRQoL of 20 conditions, thus even worse than multiple sclerosis and stroke. Overall, the same results are found after controlling for gender, age, education, and co-morbidity, including mental illness, in the OLS regression of this study, as seen in Table 5 and Fig 3.

Naturally, the study is not without limitations. The sampling method—through a patient association—raises questions of sampling bias for several reasons. Although we do not see bias in respect to age, gender, and socio-economic status compared to other studies, there might be sampling bias in respect to education. The higher education levels found contradict an American study [22], but are, however, consistent with two other American studies [1,24]. But also, multiple community-based studies suggest more severe morbidity and a higher prevalence in minority populations for ME/CFS [25]. Thus, we do not expect the patient population to be generalizable to lower social economic groups.

Furthermore, the sampling method can provide selection bias as a consequence of membership and, thus, who is answering the survey and why. We may expect unsatisfied patients, more severely affected, and/or more resourceful patients to join patient associations, as the high educational status in Table 2 indicates. On the other hand, Chu et al. suggest that clinical trials are in fact biased and underestimate HRQoL since they often do not include more severely bedridden patients, which is common among ME/CFS patients [26]. Chu et al. suggest that sampling through support groups might acquire a more accurate HRQoL than many clinical trials. If correct, we should expect a lower HRQoL estimate compared to other studies, since the ME population in the current study includes support groups as a result of their membership of the ME/CFS association.

Another issue is the use of self-reported conditions—even though strengths and limitations of self-reported study designs are shared and accepted in many other studies, several also calculating HRQoL [14,27–31]. Different diagnostic criteria of ME/CFS tend to impact the severity and thus the HRQoL of the condition [2,19]; in addition there are currently five criteria in use [32]. Hence, a limitation of this study is that it cannot distinguish between diagnostic criteria used since the condition is self-reported. Furthermore, it is well known that use of self-reported conditions can be biased [33,34], which is why a different study design should be considered. In a Danish context, national registries contain physician-reported ICD-10 diagnoses of the full population for all contact with the secondary health-care system [35]. Consequently, it was initially explored whether the physician-reported conditions from registries could be an alternative study design to the survey self-reported conditions. Nevertheless, using national hospital registries to include ME/CFS patients (ICD-10 code G93.3) only identifies 746 patients in the period of 1994–2013 of the entire population of 4,555,439 million people aged 16+ at 1/1/2013 (see S1 Table)–or just three in the health profile survey sample merged with registries (if enough registry conditions could have been merged with the survey sample, we could have estimated the EQ-5D-3L HRQoL for the physician-reported conditions using the Population Health Profile sample alone). This equals a prevalence of 0.014% of the full population—and is far from the prevalence of 0.2–0.4 reported in other studies [3]. In conclusion, it is not possible to achieve a representative useful ME/CFS population based on hospital and doctor-reported ICD-10 condition extracted from national registries. This unexpected result may have several explanations. One is that health professionals internationally have difficulties and disagreements defining and diagnosing the condition [21]. In addition, the existing international definitions are mostly not used in Denmark due to a regional reclassification of ME/CFS and other diagnoses to ‘somatization’, ‘functional somatic syndromes’, and ‘bodily distress disorder’ [36,37]. This is done despite much research as well as newer American guidelines of ME/CFS diagnosis—in coherence with WHO’S ICD-10 standards—distinguish ME/CFS (G933) from somatoform disorders (F45) and other “psychogenic” clinical explanations of the condition [38]. The above issues also underline the fact that HRQoL of Danish patients is difficult to acquire without also including patient associations in Denmark in the sampling design.

Nevertheless, in respect of the definitional and aetiological discussion of ME/CFS [5,7,32,38–41], we also tested if there were a difference in the impact on the HRQoL on the unadjusted HRQoL from mental conditions, as somatoform conditions is a smaller part off, by excluding all mental conditions from the sample (n = 24), However, exclusion of mental conditions did not impact the results substantially as the mean merely increased by 0,04 (S2 table). Also, since mental conditions by current definitions are distinct from ME/CFS by newest diagnostic guidelines and recommendation [38] and ICD-10 standards, and since several studies have identified mental conditions as a part of the ME/CFS population [25,40,42], mental conditions was not excluded from the current study.

An indirect way to assess the strengths and precision of the present study’s results regarding the EQ-5D-3L estimates is by comparison to existing studies. Even though the different national EQ-5D tariff scores are difficult to compare, the only other EQ-5D study by Myers and Wilks shows a mean score of 0.56 and a median score of 0.69 [9]. Both mean and median scores are slightly higher than in the current study, but the population mean is too (0.91). Even so, the ratios between the estimates and population means reveal that the HRQoL of the current study is still slightly lower than the calculated ratio of the Myers and Wilks study (mean ratio of 0.566 versus Myers and Wilks of 0.615). Other newer studies also indicate similar and considerable low HRQoL for ME/CFS patients using other generic HRQoL measures [1,2,9,19]. Nacul et al. also show that ME/CFS has the lowest SF-36 HRQoL compared to other conditions such as cancer, depression, diabetes, heart and lung disease, rheumatoid arthritis and osteoarthritis. Also, calculated ratios of both the mean of the physical and mental SF-36 dimensions to the population mean are similar to the present study (ratio of physical dimension is 0.54 and mental dimension is 0.68). Thus, we do not find other studies that contradict the current results. But we do not know whether the slightly lower HRQoL of this study is biased by the sampling method, or whether it is actually closer to the true estimates as Chu et al. indirectly suggests, since a patient association is expected to include more of the missing severely affected patients not usually included in trials and common study designs.

Finally, an important strength of the current study is the adjusted analysis. None of the referenced studies conduct an adjusted regression analysis taking age, gender, education, or, perhaps most importantly, co-morbidity into account. One of the benefits of doing so is ‘extracting’ the effect of the HRQoL from other conditions, leaving the pure utility effect of ME/CFS estimated. The adjusted analysis indicates that HRQoL of MF/CFS patients is different from, and thus not a proxy, of other conditions—including mental illnesses. This is important, as one of the cornerstones in the aetiological discussion concerning this disease is whether it should be regarded basically as a psychiatric or somatic disease entity [5].

However, further and thorough clinical studies are needed to confirm the statistical indications to be able to draw more firm conclusions on the aetiology. In this respect, the major strength of this study is the finding regarding the very poor HRQoL of a relatively large group of patients, regardless of the aetiology. This alone should have implications for clinicians, researchers and policymakers in relation to the ongoing debate and treatments options offered.

## Conclusion

The study provides the first Danish EQ-5D-3L preference scores for ME/CFS patients as well as the newest EQ-5D-3L estimates for use internationally in health economics, health-care planning and research. It is found that ME/CFS in this study has a major impact on the patients’ HRQoL. In conclusion, the EQ-5D-3L-based HRQoL of ME/CFS is significantly lower than the population mean. In fact, the ME/CFS HRQoL in this study was the lowest of all the compared conditions in both the unadjusted analysis and in the adjusted regression analysis. The adjusted regression analysis indicates that the HRQoL of MF/CFS is not a proxy of the 18 other included conditions. Finally, the ME/CFS estimates are also slightly lower than those of older studies. Possible selection bias due to the sampling method was discussed, and it is recommended that further studies are conducted to explore and exclude the possible sampling issues of this study.

## Supporting Information

S1 File Survey questions as published (Danish and English). (PDF)

S2 File Adjusted OLS regression model with interactions. (PDF)

S3 File ME/CFS sample of 112 respondents. Full STATA dataset with imputed variables. (DTA)

S1 Table Data-extracted frequencies of ME/CFS hospital treated patients from 1994–2013, ICD-10 code G93.3 based on a Danish full population and sample data. (PDF)

S2 Table Unadjusted means of ME/CFS patients without mental conditions. (PDF)
