# Supplementary material for: The Health-Related Quality of Life for Patients with Myalgic Encephalomyelitis / Chronic Fatigue Syndrome (ME/CFS)
Source: PLoS One. 2015 Jul 6;10(7):e0132421. doi: 10.1371/journal.pone.0132421 (PMC4492975; doi:10.1371/journal.pone.0132421)
Supplement: S1 File — (PDF) [file pone.0132421.s001.pdf]

S1 File. Survey questions as published (Danish and English).

(Appendix 1)

- Appendix 1a – survey questions as published (English translated version)
- Appendix 1b – survey questions as published (Danish original version)

Appendix 1a – survey questions as published (English translated version)

How to fill out the survey

First print out a questionnaire. Next, fill it, as described below.
Once you have completed it, please send it to:

ME Foreningen, Rådhustorvet 1, 1, 3520 Farum.

Please use a black or blue pen or thin marker.

The answers should be written so that all numbers and crosses should be easy to interpret as shown in the following examples:

	CORRECT.	WRONG
Please set clear **cross.**		
If a field is filled out incorrectly, shade the box and set a new cross in the correct box.		
Numbers are written within the fields of the box.	1,2	2
Numbers are corrected by striking out the wrong number and write the correct number above.		

Quality of Life Survey - ME 2013

By placing a tick in one box in each group below, please indicate which statements best describe your own health state today.

Mobility

I have no problems in walking about ❑

I have some problems in walking about ❑

I am confined to bed ❑

Self-Care

I have no problems with self-care ❑

I have some problems washing or dressing myself ❑

I am unable to wash or dress myself ❑

Usual Activities (*e.g. work, study, housework, family or leisure activities)*

I have no problems with performing my usual activities ❑

I have some problems with performing my usual activities ❑

I am unable to perform my usual activities ❑

Pain/Discomfort

I have no pain or discomfort ❑

I have moderate pain or discomfort ❑

I have extreme pain or discomfort ❑

Anxiety/Depression

I am not anxious or depressed ❑

I am moderately anxious or depressed ❑

I am extremely anxious or depressed ❑

To help people say how good or bad a health state is, we have drawn a scale (rather like a thermometer) on which the best state you can imagine is marked 100 and the worst state you can imagine is marked 0.

We would like you to indicate on this scale how good or bad your own health is today, in your opinion. Please do this by drawing a line from the box below to whichever point on the scale indicates how good or bad your health state is today.

Your own

health stata

today

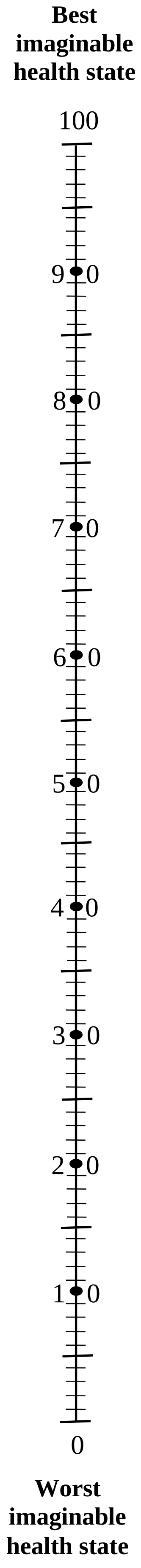

Long-term illness and after-effects

Do you have any long-term illness, long-term after effects of injuries, disability or other long-term illness?

(Please only put one X)

Yes .... ☐ 1

No .... ☐ 2

For each of the following diseases and health problems, please indicate whether you have it now or have had it earlier. If you've had it before, please also indicate whether you have after effects.

	(Only one X in every line)				*(Only one X in every line)* **If you have had it earlier: Do you still have after effects?**	
	**No, that I <u>never</u> have had**	**Yes, that I have <u>now</u>**	**Yes, that I have had <u>previous</u>**		**Yes**	**No**
ME / CFS	☐1	☐2	☐3	→	☐1	☐2
Allergy (not astma)	☐1	☐2	☐3	→	☐1	☐2
Diabetes	☐1	☐2	☐3	→	☐1	☐2
High blood pressure	☐1	☐2	☐3	→	☐1	☐2
Heart attack	☐1	☐2	☐3	→	☐1	☐2
Angina	☐1	☐2	☐3	→	☐1	☐2
Stroke	☐1	☐2	☐3	→	☐1	☐2
Chronic bronchitis, emphysema, COPD (emphysema, COPD)	☐1	☐2	☐3	→	☐1	☐2
Osteoarthritis	☐1	☐2	☐3	→	☐1	☐2
Osteoarthritis	☐1	☐2	☐3	→	☐1	☐2
Osteoporosis	☐1	☐2	☐3	→	☐1	☐2
Cancer	☐1	☐2	☐3	→	☐1	☐2
Migraine or frequent headaches	☐1	☐2	☐3	→	☐1	☐2
Short term mental illness	☐1	☐2	☐3	→	☐1	☐2

(for example light depression or anxiety)						
Long term mental illness or mental disorders	☐1	☐2	☐3	→	☐1	☐2
Herniated or other back disorders	☐1	☐2	☐3	→	☐1	☐2
cataracts	☐1	☐2	☐3	→	☐1	☐2
Tinnitus	☐1	☐2	☐3	→	☐1	☐2

Such all in all …

All things considered - how satisfied or dissatisfied are you currently with life? (1 means very dissatisfied and 10 means very satisfied. 88 means "do not know")

(Please only put one x)

**Very unsatisfied**									**Very satisfied**	**Don't know**
☐	☐	☐	☐	☐	☐	☐	☐	☐	☐	☐
1	2	3	4	5	6	7	8	9	10	88

<u>Personal relationships</u>

Are you..?

Man .... ☐ 1

Women... ☐ 2

What year were you born?

Write year you were born:: 19 ☐☐

In which Region do you live..?

Capital Region .... ☐ 1

Region Zealand.. ☐ 2

Region of Southern Denmark.. ☐ 3

Central Denmark Region .... ☐ 4

North Denmark Region .... ☐ 5

Do you live alone or with others?

(You may put several X's)

I live alone	☐1
I live with my spouse or partner	☐2
I live with my parents	☐3
I live with a child / children <u>under</u> 16 years	☐4
I live with young / young people (16-20 years)	☐5
live with other adults **above** 20 years	☐6

What education do you have?

(Please only put one X)

Still goes to school	☐1
7 or fewer years of schooling	☐2
8-9 years of schooling	☐3
10-11 years of schooling	☐4
Student, HF-exam (incl. HHX, HTX)	☐5
Other (including foreign school)	☐6

Which school or youth education are you doing?

	(Please only put one X)
9. grade	☐1
10. grade	☐2
STX	☐3
HTX	☐4
HHX	☐5
Trade school basic	☐6
EUD (technical school)	☐7
HF	☐8
Other	☐9

Have you completed an education beyond basic school- or youth education?

	(Please only put one X)
No	☐1
One or more shorter courses (eg specialist worker training, labor rates, etc.)	☐2
Vocational education / skilled (eg, office or shop assistants, hairdressers, bricklayers, medical secretary, social and health / assistant, farmer)	☐3
Short education, 2-3 years (eg, market economist, police officer, laboratory technician, mechanical engineer, computer science, multimedia, catering manager, dental hygienist)	☐4
Medium education, 3-4 years (for example, school, social worker, building technician, nurse, physiotherapist, BSc, educator, bachelor)	☐5
Long higher education, more than 4 years (eg MSc, MA., Doctor, psychologist)	☐6
Other education	☐7

Are you within education?

(Please only put one X)

Yes .... ☐ 1

No .... ☐ 2

Are you employed?

(Please only put one X)

Yes .... ☐ 1

No .... ☐ 2

(Please only put one X)

**Are you:**	
Age-related retirement	☐1
Disability pensioner	☐2
A different type of pension	☐3
Pre- retirement, transitional allowance	☐4
Unemployment benefits (union)	☐5
Sickness benefit	☐6
Long term sick (3 months. Or more)	☐7
Flex-job	☐8
On social security, welfare	☐9
During rehabilitation	☐10
Other	☐11
If other, please write what: ______________________________	

You have now completed the study.
Please submit your answer to:

ME Foreningen

Rådhustorvet 1, 1

35 20 Farum

ME Association thank you for your participation which is of great value to our contact with our health politicians.

Sincerely,
Rebecca Hansen
ME Association, Chairman.

Appendix 1b – survey questions as published (Danish version)

Sådan udfylder du skemaet

Først print spørgeskemaet ud. Dernæst udfyld det, som beskrevet nedenunder.

Når du har udfyldt det, så send det til:

ME Foreningen, Rådhustorvet 1, 1, 3520 Farum.

Brug venligst en **sort** eller **blå kuglepen** eller **tynd filtpen.**

Svarene skal skrives ind, så alle tal og kryds skal være nemme at tolke som vist i nedenstående eksempler:

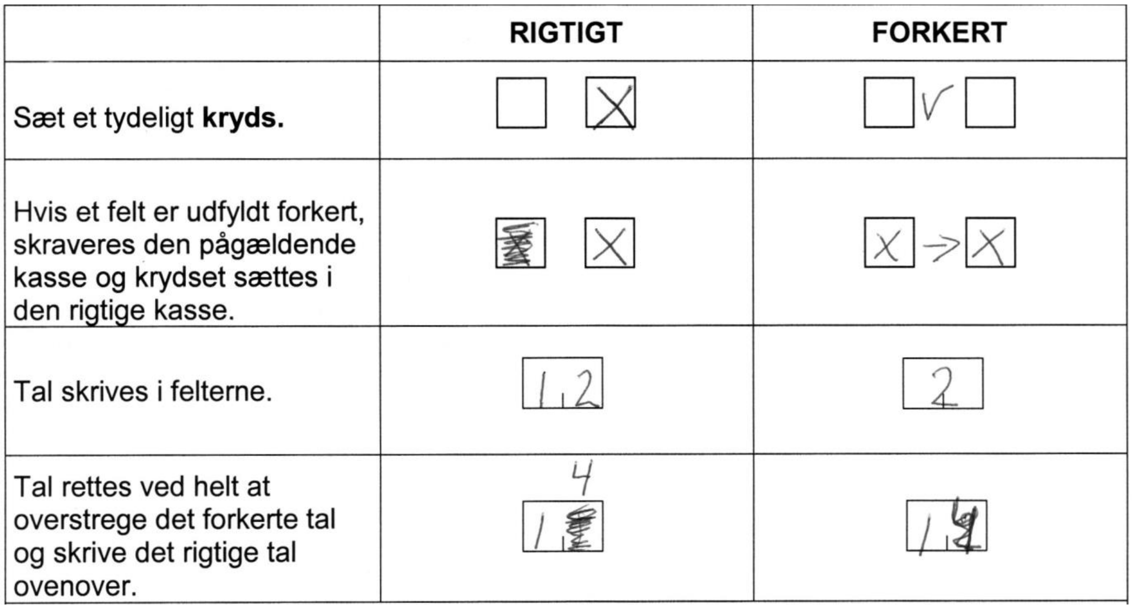

	RIGTIGT	FORKERT
Sæt et tydeligt **kryds.**		
Hvis et felt er udfyldt forkert, skraveres den pågældende kasse og krydset sættes i den rigtige kasse.		
Tal skrives i felterne.		
Tal rettes ved helt at overstrege det forkerte tal og skrive det rigtige tal ovenover.		

Livskvalitetsundersøgelse ME 2013

Angiv, ved at sætte kryds i én af kasserne i hver gruppe, hvilke udsagn, der bedst beskriver din helbredstilstand i dag.

Bevægelighed

Jeg har ingen problemer med at gå omkring ❑1

Jeg har nogle problemer med at gå omkring ❑2

Jeg er bundet til sengen ❑3

Personlig pleje

Jeg har ingen problemer med min personlige pleje ❑1

Jeg har nogle problemer med at vaske mig eller klæde mig på ❑2

Jeg kan ikke vaske mig eller klæde mig på ❑3

Sædvanlige aktiviteter *(fx.arbejde, studie, husarbejde, familie- eller fritidsaktiviteter)*

Jeg har ingen problemer med at udføre mine sædvanlige aktiviteter ❑1

Jeg har nogle problemer med at udføre mine sædvanlige aktiviteter ❑2

Jeg kan ikke udføre mine sædvanlige aktiviteter ❑3

Smerter/ubehag

Jeg har ingen smerter eller ubehag ❑1

Jeg har moderate smerter eller ubehag ❑2

Jeg har ekstreme smerter eller ubehag ❑3

Angst/depression

Jeg er ikke ængstelig eller deprimeret ❑1

Jeg er moderat ængstelig eller deprimeret ❑2

Jeg er ekstremt ængstelig eller deprimeret ❑3

Bedst
tænkelige
helbredstilstand

For at hjælpe folk med at sige, hvor god eller dårlig en helbredstilstand er, har vi tegnet en skala (næsten ligesom et termometer), hvor den bedste helbredstilstand du kan forestille dig er markeret med 100, og den værste helbredstilstand du kan forestille dig er markeret med 0.

Vi beder dig angive på denne skala, hvor god eller dårlig du mener din egen helbredstilstand er i dag. Angiv dette ved at tegne en streg fra kassen nedenfor til et hvilket som helst punkt på skalaen, der viser, hvor god eller dårlig din helbredstilstand er i dag.

Din egen

helbredstilstand

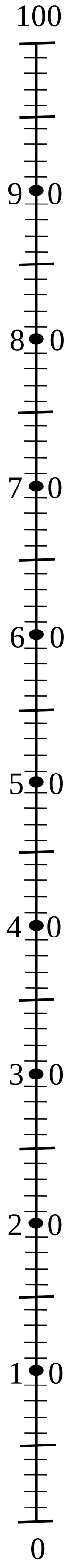

Værst

tænkelige
helbredstilstand

Langvarige sygdomme og eftervirkninger

Har du nogen langvarig sygdom, langvarig eftervirkning af skade, handicap eller anden langvarig lidelse?

(Kun ét X)

Ja .... ☐ 1

Nej .... ☐ 2

For hver af de følgende sygdomme og helbredsproblemer bedes du angive, om du har den nu eller har haft den tidligere. Hvis du har haft den tidligere, bedes du også angive, om du har eftervirkninger.

	(Sæt ét X i hver linje)				*(Sæt ét X i hver linje)*	
	**Nej, det har jeg aldrig haft**	**Ja, det har jeg nu**	**Ja, det har jeg haft tidligere**		**Hvis du har haft det tidligere: Har du stadig eftervirkninger? Ja**	**Nej**
ME / CFS	☐1	☐2	☐3	→	☐1	☐2
Allergi (ikke astma)	☐1	☐2	☐3	→	☐1	☐2
Sukkersyge (diabetes)	☐1	☐2	☐3	→	☐1	☐2
Forhøjet blodtryk	☐1	☐2	☐3	→	☐1	☐2
Blodprop i hjertet	☐1	☐2	☐3	→	☐1	☐2
Hjertekrampe	☐1	☐2	☐3	→	☐1	☐2
Hjerneblødning, blodprop i hjernen	☐1	☐2	☐3	→	☐1	☐2
Kronisk bronkitis, for store lunger, rygerlunger (emfysem, KOL)	☐1	☐2	☐3	→	☐1	☐2
Slidgigt	☐1	☐2	☐3	→	☐1	☐2
Leddegigt	☐1	☐2	☐3	→	☐1	☐2
Knogleskørhed (osteoporose)	☐1	☐2	☐3	→	☐1	☐2
Kræft	☐1	☐2	☐3	→	☐1	☐2
Migræne eller hyppig hovedpine	☐1	☐2	☐3	→	☐1	☐2
Forbigående psykisk lidelse (f.eks. let depression eller angst)	☐1	☐2	☐3	→	☐1	☐2

Vedvarende psykisk sygdom eller mentale forstyrrelser	☐1	☐2	☐3	→	☐1	☐2
Diskusprolaps eller andre rygsygdomme	☐1	☐2	☐3	→	☐1	☐2
Grå stær	☐1	☐2	☐3	→	☐1	☐2
Tinnitus	☐1	☐2	☐3	→	☐1	☐2

Sådan alt i alt…

Alt taget i betragtning - hvor tilfreds eller utilfreds er du for tiden med <u>livet</u>? (1 betyder meget utilfreds og 10 betyder meget tilfreds. 88 betyder "ved ikke")

(Sæt kun ét X
)

**Meget utilfreds**									**Meget Tilfreds**	**Ved ikke**
☐	☐	☐	☐	☐	☐	☐	☐	☐	☐	☐
1	2	3	4	5	6	7	8	9	10	88

Personlige forhold

Er du..?

Mand .... ☐ 1

Kvinde .... ☐ 2

Hvilket år er du født?

Skriv år du er født: 19 ☐☐

I hvilken Region bor du..?

Region Hovedstaden .... ☐ 1

Region Sjælland .... ☐ 2

Region Syddanmark	☐ 3
Region Midtjylland	☐ 4
Region Nordjylland..	☐ 5

Bor du alene eller sammen med andre?

(Sæt gerne flere X'er)

	(Kun ét X)
Jeg bor alene	☐1
Jeg bor sammen med ægtefælle eller samlever	☐2
Jeg bor sammen med forældre	☐3
Jeg bor sammen med barn/børn **under** 16 år	☐4
Jeg bor sammen med ung/unge (16-20 år)	☐5
Jeg bor sammen med andre voksne **over** 20 år	☐6

Hvilken skoleuddannelse har du?

	(Kun ét x)
Går stadig i skole	☐1
7 eller færre års skolegang	☐2
8-9 års skolegang	☐3
10-11 års skolegang	☐4
Studenter-, HF-eksamen (inkl. HHX, HTX)	☐5
Andet (herunder udenlandsk skole)	☐6

Hvilken skole- eller ungdomsuddannelse er du i gang med?
(Kun ét X)

	(Kun ét x)
9. klasse	☐1
10. klasse	☐2
STX	☐3
HTX	☐4

HHX	☐5
Handelsskolens grunduddannelse	☐6
EUD (teknisk skole)	☐7
HF	☐8
Andet	☐9

Har du fuldført en uddannelse udover en skole- eller ungdomsuddannelse?

	(Kun ét x)
Nej	☐1
Et eller flere kortere kurser (fx specialarbejderkurser, arbejdsmarkedskurser m.v.)	☐2
Erhvervsfaglig uddannelse/faglært (fx kontor- eller butiksassistent, frisør, murer, lægesekretær, social- og sundhedshjælper/assistent, landmand)	☐3
Kort videregående uddannelse, 2-3 år (fx markedsøkonom, politibetjent, laborant, maskintekniker, datamatiker, multimediedesigner, økonoma, tandplejer)	☐4
Mellemlang videregående uddannelse, 3-4 år (fx folkeskolelærer, socialrådgiver, bygningskonstruktør, sygeplejerske, fysioterapeut, diplomingeniør, pædagog, bachelor)	☐5
Lang videregående uddannelse, mere end 4 år (fx civilingeniør, cand.mag., læge, psykolog)	☐6
Anden uddannelse	☐7

Er du under uddannelse?

Ja .... ☐ 1

Nej .... ☐ 2

Er du i arbejde?

Ja .... ☐ 1

Nej .... ☐ 2

Sæt kun 1 kryds:

**Er du:**	
Alderspensionist	☐1
Førtidspensionist	☐2
Anden form for pension	☐3
Efterlønsmodtager, overgangsydelse	☐4

Dagpenge	☐5
Sygedagpenge	☐6
Langtidssyg (3 mdr. eller mere)	☐7
Fleskjob	☐8
På kontanthjælp, bistandshjælp	☐9
Under revalidering	☐10
Andet	☐11
Hvis andet, skriv hvad: ____	

Du har nu afsluttet undersøgelsen.

Du bedes indsende din besvarelse til

ME Foreningen

Rådhustorvet 1, 1

35 20 Farum

ME Foreningen takker for din deltagelse som er af stor værdi for vores kontakt til bl.a. sundhedspolitikere.

Med venlig hilsen

Rebecca Hansen

ME Foreningen, formand.

Appendix 2. Frequencies data-extraction of ME/CFS hospital treated patients from 1994-2013, ICD-10 code G93.3 based on a Danish full population and sample data

Full Population yearly incidence for diagnosis code G93.3 (ME/CFS)

year	Frequency	Cumulative Frequency
1994	44	44
1995	46	90
1996	46	136
1997	63	199
1998	53	252
1999	40	292
2000	27	319
2001	24	343
2002	37	380
2003	43	423
2004	43	466
2005	30	496
2006	32	528
2007	27	555
2008	26	581
2009	31	612
2010	24	636
2011	30	666
2012	35	701
2013	44	745

Only respondents of the North Denmark Health Survey sample of 2010, merged with hospital data, yearly incidence for diagnosis code G93.3:

year	Frequency	Cumulative Frequency
**1998**	2	2
**2005**	1	3

Source: The Danish National Patient Register, data extracted from Statistics Denmark Researcher Servers.
