# Supplementary material for: The Health-Related Quality of Life for Patients with Myalgic Encephalomyelitis / Chronic Fatigue Syndrome (ME/CFS)
Source: PLoS One. 2015 Jul 6;10(7):e0132421. doi: 10.1371/journal.pone.0132421 (PMC4492975; doi:10.1371/journal.pone.0132421)
Supplement: S2 File — (PDF) [file pone.0132421.s002.pdf]

```
                                          ___  ____  ____  ____  ____(R)
                                         /__    /   ____/   /   ____/
                                        ___/   /   /___/   /   /___/
                                          Statistics/Data Analysis


  ___  ____  ____  ____  ____ (R)
 /__    /   ____/   /   ____/
___/   /   /___/   /   /___/   12.1   Copyright 1985-2011 StataCorp LP
  Statistics/Data Analysis            StataCorp
                                      4905 Lakeway Drive
     MP - Parallel Edition            College Station, Texas 77845 USA
                                      800-STATA-PC        http://www.stata.com
                                      979-696-4600        stata@stata.com
                                      979-696-4601 (fax)

Single-user 4-core Stata perpetual license:
       Serial number:  50120574516
         Licensed to:  SAMF-IT
                       Aalborg Universitet

Notes:
      1.  (/v# option or -set maxvar-) 5000 maximum variables

1 . do "C:\Users\hvidberg\AppData\Local\Temp\STD01000000.tmp"

2 . cd C:\PHD_temp\DATAE
C:\PHD_temp\DATAE

3 . use me_sp2010_imp.dta, clear

4 .
end of do-file

5 . do "C:\Users\hvidberg\AppData\Local\Temp\STD01000000.tmp"

6 . mi unregister st_vgt_kau

7 .
end of do-file

8 . do "C:\Users\hvidberg\AppData\Local\Temp\STD01000000.tmp"

9 . set more off
```

```
. mi estimate: svy: regress eq_tto_imp i.me##i.gender i.me##c.age i.me##i.education i.me##i.allergy i.me#
> #i.diabetes i.me##i.high_blood_pressure i.Heart_attack i.me##i.Angina_pectoris i.me##i.stroke
> i.me##i.Lung_diseases_COPH_others i.me##i.Osteoarthritis i.me##i.Rheumatoid_arthritis i.me##i.Osteoporosis
i.me##i.cancer
> i.me##i.migraine_headegg i.me##i.all_mental_conditions i.me##i.Herniated_disc_or_other_back_cond
i.me##i.cataracts       > i.me##i.tinnitus

Multiple-imputation estimates                   Imputations       =         20
Survey: Linear regression                       Number of obs     =      23504

Number of strata  =         1                   Population size   = .99939588
Number of PSUs    =     23504
                                                Average RVI       =     0.4286
                                                Largest FMI       =     0.8439
                                                Complete DF       =      23503
DF adjustment:   Small sample                   DF:     min       =      27.20
                                                        avg       =    1456.72
                                                        max       =   10003.55
Model F test:       Equal FMI                   F(  42, 6265.7)   =      99.70
Within VCE type:   Linearized                   Prob > F          =     0.0000

-----------------------------------------------------------------------------------------------
                   eq_tto_imp |      Coef.   Std. Err.      t    P>|t|     [95% Conf. Interval]
------------------------------+----------------------------------------------------------------
                         1.me |  -.4980455   .1654301    -3.01   0.003    -.8223552   -.1737358
                     1.gender |   .0045614   .0024751     1.84   0.065    -.0002907    .0094135
                              |
                    me#gender |
                         1 1  |   .0635242   .0935568     0.68   0.497    -.1199058    .2469541
                              |
                          age |  -.0006105   .0000916    -6.67   0.000    -.0007903   -.0004308
                              |
                     me#c.age |
                           1  |   .0000294   .0030106     0.01   0.992    -.0058751     .005934
                              |
                    education |
                           2  |   .0174533    .003213     5.43   0.000     .0111478    .0237588
                           3  |   .0351088   .0036925     9.51   0.000     .0278596    .0423579
                           4  |   .0386155   .0047835     8.07   0.000     .0292362    .0479948
                              |
                 me#education |
                         1 2  |   .2174728   .1092788     1.99   0.047     .0029906    .4319549
                         1 3  |   .2228863   .1142987     1.95   0.052    -.0017117    .4474843
                         1 4  |   .1184169   .1500694     0.79   0.430    -.1760982     .412932
```

1.allergy	-.0095699	.0035452	-2.70	0.007	-.0165369	-.002603
me#allergy						
1 1	-.095575	.0673847	-1.42	0.156	-.2276822	.0365321
1.diabetes	-.0204928	.0083669	-2.45	0.015	-.0370062	-.0039795
me#diabetes						
1 1	.1077051	.1581224	0.68	0.497	-.2059894	.4213996
1.high_blood_pressure	-.0090695	.0038579	-2.35	0.019	-.0166444	-.0014947
me#high_blood_pressure						
1 1	.1525823	.0751352	2.03	0.043	.0051583	.3000063
1.Heart_attack	.0205157	.0209397	0.98	0.336	-.0224342	.0634656
1.Angina_pectoris	-.0485958	.0179546	-2.71	0.008	-.0840515	-.01314
me#Angina_pectoris						
1 1	.034324	.0848839	0.40	0.686	-.132455	.2011031
1.stroke	-.1366055	.0201545	-6.78	0.000	-.1767429	-.0964681
me#stroke						
1 1	-.1379463	.1817028	-0.76	0.448	-.4945322	.2186396
1.Lung_diseases_COPH_others	-.0353441	.0088876	-3.98	0.000	-.0528212	-.0178669
me#Lung_diseases_COPH_others						
1 1	.1010685	.1305769	0.77	0.440	-.157044	.359181
1.Osteoarthritis	-.0742935	.0039907	-18.62	0.000	-.0821326	-.0664544
me#Osteoarthritis						
1 1	.1661346	.0707131	2.35	0.019	.0273654	.3049038
1.Rheumatoid_arthritis	-.0540127	.006605	-8.18	0.000	-.0669699	-.0410556
me#Rheumatoid_arthritis						
1 1	.0737035	.0935972	0.79	0.431	-.1099513	.2573583
1.Osteoporosis	-.0573542	.0121246	-4.73	0.000	-.0814193	-.033289
me#Osteoporosis						

1 1	-.1956159	.0904009	-2.16	0.031	-.3730003	-.0182316
1.cancer	-.0287331	.0126977	-2.26	0.026	-.0538973	-.0035689
me#cancer						
1 1	.2239935	.1365229	1.64	0.101	-.0440483	.4920353
1.migraine_headegg	-.0625139	.0041629	-15.02	0.000	-.0706822	-.0543456
me#migraine_headegg						
1 1	-.0025414	.0682481	-0.04	0.970	-.1363715	.1312887
1.all_mental_conditions	-.1702654	.0051466	-33.08	0.000	-.1803606	-.1601702
me#all_mental_conditions						
1 1	-.0481383	.0716299	-0.67	0.502	-.1885798	.0923031
1.Herniated_disc_or_other_back	-.0990844	.0046966	-21.10	0.000	-.1083019	-.0898668
me#Herniated_disc_or_other_back						
1 1	.086995	.0584452	1.49	0.137	-.027644	.201634
1.cataracts	-.0027922	.009277	-0.30	0.764	-.0211554	.015571
me#cataracts						
1 1	.0117146	.1153131	0.10	0.919	-.2148429	.2382722
1.tinnitus	.0024045	.0050124	0.48	0.632	-.0074439	.0122529
me#tinnitus						
1 1	-.0473047	.0608106	-0.78	0.437	-.1666331	.0720237
_cons	.9390919	.0045187	207.82	0.000	.9302343	.9479494

NOTE: some interactions do not exist, for example Heart_attack and ME (no ME patients have had a heart atteck). Theese interactions are thus not included to ensure correct estimatation.

NOTE: original file names are translated to english in the table.
