# Supplementary material for: The Health-Related Quality of Life for Patients with Myalgic Encephalomyelitis / Chronic Fatigue Syndrome (ME/CFS)
Source: PLoS One. 2015 Jul 6;10(7):e0132421. doi: 10.1371/journal.pone.0132421 (PMC4492975; doi:10.1371/journal.pone.0132421)
Supplement: S1 Table — (PDF) [file pone.0132421.s004.pdf]

S1 Table. Data-extracted frequencies of ME/CFS hospital treated patients from 1994-2013, ICD-10 code G93.3 based on a Danish full population and sample data. (Appendix 2.)

<u>Full Population yearly incidence for diagnosis code G93.3 (ME/CFS)</u>

year	Frequency	Cumulative Frequency
1994	44	44
1995	46	90
1996	46	136
1997	63	199
1998	53	252
1999	40	292
2000	27	319
2001	24	343
2002	37	380
2003	43	423
2004	43	466
2005	30	496
2006	32	528
2007	27	555
2008	26	581
2009	31	612
2010	24	636
2011	30	666
2012	35	701
2013	44	745

Only respondents of the North Denmark Health Survey sample of 2010, merged with hospital data, yearly incidence for diagnosis code G93.3:

year	Frequency	Cumulative Frequency
**1998**	2	2
**2005**	1	3

Source: The Danish National Patient Register, data extracted from Statistics Denmark Researcher Servers.
