# Supplementary material for: The Health-Related Quality of Life for Patients with Myalgic Encephalomyelitis / Chronic Fatigue Syndrome (ME/CFS)
Source: PLoS One. 2015 Jul 6;10(7):e0132421. doi: 10.1371/journal.pone.0132421 (PMC4492975; doi:10.1371/journal.pone.0132421)
Supplement: S2 Table — (PDF) [file pone.0132421.s005.pdf]

S2 Table.

Mean of ME/CFS with and without mental conditions excluded. Complete cases.

	Mean	Std. Err.	[95% Conf. Interval]	n
Sample I – ME/CFS with mental conditions	0.469	0.031	[0.407 - 0.530]	103
Sample II – ME/CFS without mental conditions	0.509	0.033	[0.444 - 0.575]	79

Two-sample t test with equal variances*

Group	Obs	Mean	Std. Err.	Std. Dev.	[95% Conf. Interval]
1	103	0.4687379	0.0313283	0.3179472	[0.4065984 ; 0.5308773]
2	79	0.5095696	0.0326525	0.2902219	[0.4445635 ; 0.5745758]
combined	182	0.4864615	0.0226872	0.3060676	[0.4416961 ; 0.531227]
diff		-0.0408318	0.0458002		[-0.1312061 ; 0.0495426]

diff = mean(1) - mean(2) **t = -0.8915**

Ho: diff = 0 **degrees of freedom = 180**

Ha: diff < 0 **Ha: diff != 0** **Ha: diff > 0**

Pr(T < t) = 0.1869 **Pr(|T| > |t|) = 0.3738** **Pr(T > t) = 0.8131**

---

* NOTE: since the two samples are overlapping and not independent, the ttest is not an optimal test of mean difference, since the assumption of independence is violated. However, to the authors' knowledge, there is no consensuses of which test is the best to apply in this case.
